# Efficient calculation of the quasi-static electrical potential on a tetrahedral mesh and its implementation in STEPS

**DOI:** 10.3389/fncom.2013.00129

**Published:** 2013-10-29

**Authors:** Iain Hepburn, Robert Cannon, Erik De Schutter

**Affiliations:** ^1^Computational Neuroscience Unit, Okinawa Institute of Science and TechnologyOnna-son, Japan; ^2^Theoretical Neurobiology, University of AntwerpAntwerp, Belgium; ^3^Textensor LimitedEdinburgh, UK

**Keywords:** 3D electrical potential, tetrahedral meshes, membrane potential, complex morphology, spatial stochastic simulation, multiscale simulation, efficient node ordering

## Abstract

We describe a novel method for calculating the quasi-static electrical potential on tetrahedral meshes, which we call E-Field. The E-Field method is implemented in STEPS, which performs stochastic spatial reaction-diffusion computations in tetrahedral-based cellular geometry reconstructions. This provides a level of integration between electrical excitability and spatial molecular dynamics in realistic cellular morphology not previously achievable. Deterministic solutions are also possible. By performing the Rallpack tests we demonstrate the accuracy of the E-Field method. Efficient node ordering is an important practical consideration, and we find that a breadth-first search provides the best solutions, although principal axis ordering suffices for some geometries. We discuss potential applications and possible future directions, and predict that the E-Field implementation in STEPS will play an important role in the future of multiscale neural simulations.

## 1. Introduction

In computational neuroscience, up until now studies of the electrical behavior of cells and networks have not often included detailed biochemical signaling network components. Vice-versa, studies of molecular systems have usually not taken into account the electrical excitablity of the cellular membranes that surround them. While many important advances in our understanding of neural systems have been made by this approach, future studies are expected to focus more and more on the interaction across different spatial and temporal scales exploring the impact that different systems have on each other, an approach often termed multiscale modeling. Although adding a new level of complexity to simulations, multiscale modeling is expected to play a vital role in the future of computational neuroscience (Djurfeldt et al., 2010; Bhalla, 2011; Anwar et al., 2013).

Developing tools that can perform seamless integration across different spatial and temporal scales is a challenging task. In cellular simulations this amounts to connecting the electrical excitability of the cell with reaction-diffusion models of biochemical networks. Furthermore, computations must be as efficient as possible so that no component forms a bottleneck, yet must not over-simplify any component so as to ensure no significant loss of accuracy. An increasing number of simulators have been making strides toward this goal, including MOOSE (Ray and Bhalla, 2008) and GENESIS 3 (Cornelis et al., 2012). Present approaches often involve integrating two or more simulators: for example, one to perform the whole-cell electrical calculations and another carrying out reaction-diffusion calculations (Brandi et al., 2011), which already open up a wealth of potential applications. However, many simulators offer only limited morphological resolution often based on connected cylinders. There may be occasions where accurate morphological representation of complex cellular geometry below the μm scale is necessary alongside a tighter integration between the electrical and molecular systems, which is the motivation for this work. One clear example where such simulations are potentially advantageous is where a molecular species carrying a significant current across the membrane also acts as an important signaling molecule, of which one important example is calcium. When such signals are highly localized [which can often be the case (Fakler and Adelman, 2008)] and are strongly influenced by morphology (Santamaria et al., 2006; Anwar et al., 2013) a detailed spatial description of the components that are important within both the electrical and the molecular scales, namely voltage-dependent channels and signaling ions, may be vital. Furthermore, ionic channel currents are often better described by the GHK equation (Goldman, 1943; Hodgkin and Katz, 1949), and may be best modeled also as a stochastic process (Mak and Webb, 1997). These considerations together point to the need for software capable of accurate computation of electrical potential in realistic morphologies tightly coupled with a detailed spatial description of voltage-dependent channels, their transported ions and other important signaling molecules, with a full stochastic account of the interactions.

We describe a novel method we term E-Field, which performs electrical potential calculation in complex 3D morphologies represented by tetrahedral meshes, which are far more suitable for describing complex morphology than cubic meshes (Hepburn et al., 2012). E-Field is integrated in STEPS (Hepburn et al., 2012) alongside complementary components such as voltage-dependent transitions and channel currents, and tightly integrated with spatial reaction-diffusion computations based on Gillespie's SSA (Gillespie, 1977). We demonstrate accuracy and optimization efforts, describe potential applications and discuss future expansions on this groundwork.

## 2. Materials and methods

### 2.1. E-field: the tetrahedral mesh potential calculation in STEPS

The evolution of the electric field on the tetrahedral mesh is solved as a set of simultaneous difference equations for internal points and differential equations for capacitative surface elements. Each edge in the mesh gives rise to one equation and a sparse matrix method is used to compute the changes in potential over a timestep. We first show how the difference equations on the mesh are derived from the quasi-static Maxwell equations and then briefly describe the matrix method used to solve them.

#### 2.1.1. Reduction of maxwell's equations for neural tissue

For fixed and moving charges as are present in small sections of a neuron, the evolution of the electric and magnetic field is governed by the Maxwell equations. However, on the timescales of interest here, where the frequency of changes is well below the MHz range, the coupling of the magnetic field to the electrical field, which gives rise to electromagnetic waves, can be neglected (Plonsey, 1969; Nunez and Srinivasan, 2006).

Under these conditions, the system is governed by the quasi-static Maxwell equations which can be written as:

(1) $$\nabla \cdot J+\frac{\partial \rho }{\partial t}=0$$

(2) ∇·D=ρ

where *J* is the current density, *ρ* is the charge density, *t* is time and *D* is the electrical displacement. Experimentally, for most materials the quantities *J* and *D* are related to the electric field by the constitutive relations:

(3) J=σE

and

(4) D=ϵE

where σ is the conductivity, and ϵ is the permittivity.

The combination of Equations (1) and (3) expresses the conservation of charge, and Equations (2) and (4) form Gauss' law which implies that the electric field can be expressed as the gradient of the electrical potential, Φ:

(5) E=∇Φ.

From an electrical perspective, neural tissue is composed of two main materials: the cytoplasm which contains many freely moving charges and hence a relatively high conductivity, σ, and the membrane which is effectively an insulator with a very low conductivity. The cytoplasmic conductivity prevents the build-up of static charge, so, from Equations (1), (3), and (5), the potential there satisfies

(6) $$\nabla^{2}\Phi =0.$$

In principle, Equations (1) to (5) could also be used to compute the field within the membrane in terms of ϵ, σ and the membrane dimensions. In practice, this is not a useful approach because the quantities required are not well-determined. These equations do, however, give the form of the equation that governs the membrane through the standard analysis of a thin plate capacitor. This introduces a new quantity, *C*_*spec*_, the specific capacitance, such that Φ satisfies

(7) $$C_{spec}A\frac{\partial \Phi }{\partial t}+I=0$$

where *I* is the current flowing perpendicular to a section of membrane of area *A*. Experiments yield a value of *C*_*spec*_ ≈ 1μ*Fcm*^−2^.

Assuming that the segment of neurite under study is located in an earthed bath, we can take Φ = 0 outside the structure. The governing equations for the system are then Equation (6) inside the structure and (7) on the surface.

#### 2.2.2. Formulation of difference equations on a tetrahedral mesh

In order to compute the electric field numerically, the structure under study can be represented by a mesh with the potential, Φ, to be determined at the vertices of the mesh. Assuming that Φ varies linearly within each tetrahedron, the value at any point inside can be determined by linear interpolation. The first step is to divide each tetrahedron up and associate the charge inside it with one of its vertices. In effect, this creates a new set of elements centered around a vertex which will form the set of solution volumes with a one-to-one correspondence to the vertices. Equal areas of a triangle can be obtained by cuts joining the center of mass to the midpoint of each side and a similar result holds for splitting up a tetrahedron. The charge associated with a vertex is then the integral of the charge density over the associated volume. Following from (2), by Gauss' law this integral can be replaced by the integral of the normal component of the gradient of the charge density, *E*, over the surface of the volume:

(8) $$\int_{element}\rho dV=\int_{surface}E\cdot ndS$$

where *n* is the unit vector perpendicular to the surface *S*.

Consider a point *p* in the mesh surrounded by tetrahedra and one such tetrahedron with corners at *p*, *a*, *b*, and *c* where *a*, *b* and *c* form a right-handed set. Let a, b, and c be the vectors from *p* to *a*, *b*, and *c*, respectively.

The potential at a point (x, y, z) within the tetrahedron can be written as

(9) $$\Phi =\Phi_{p}+(\alpha ,\beta ,\gamma )\left(\begin{array}{c} x\\ y\\ z \end{array}\right)$$

for unknowns α, β, and γ. By coordinate transformation, the potentials at the vertices, Φ_*a*_, Φ_*b*_, and Φ_*c*_ satisfy:

(10) $$\left(\begin{array}{c} \Phi_{a}\\ \Phi_{b}\\ \Phi_{c} \end{array}\right)=(\alpha ,\beta ,\gamma )\left[\begin{array}{ccc} a_{x} & b_{x} & c_{x}\\ a_{y} & b_{y} & c_{y}\\ a_{z} & b_{z} & c_{z} \end{array}\right]$$

where *a*_*x*_ etc are the components of the vectors a etc.

This can be written as a matrix equation:

$$\left(\begin{array}{c} \Phi_{a}\\ \Phi_{b}\\ \Phi_{c} \end{array}\right)=M\left(\begin{array}{c} \alpha \\ \beta \\ \gamma \end{array}\right)$$

where *M* is the transpose of the matrix in Equation (10). The inverse of M then gives α, β, and γ in terms of the potentials:

(11) $$\left(\begin{array}{c} \alpha \\ \beta \\ \gamma \end{array}\right)=M^{-1}\left(\begin{array}{c} \Phi_{a}\\ \Phi_{b}\\ \Phi_{c} \end{array}\right).$$

The combination of Equations (9) and (11) now gives the potential anywhere in the tetrahedron in terms of the geometrical constants and the potentials at the vertices.

**Figure 1:**
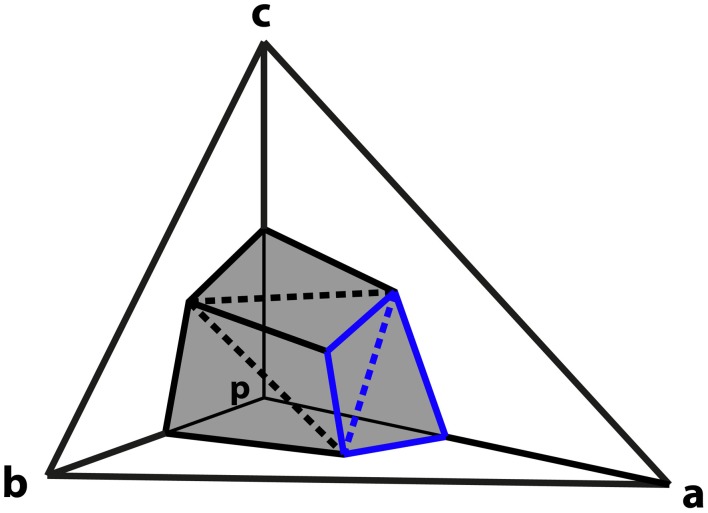
**Derivation of vertex coupling constants in tetrahedral mesh**. A tetrahedron is comprised of vertices p, a, b, and c. The surface of the element centered on p that intersects tetrahedron formed by a, b, and c consists of six triangles (shaded). The triangles described by Equations (12) and (13) in the text are highlighted in blue. Dashed lines separate triangles that are in the same plane.

The next step is to derive an expression for the charge associated with a particular vertex in terms of the potentials of the neighboring vertices. The part of the surface of the element centered on *p* that intersects tetrahedron *a−b−c* is composed of six triangles as shown in Figure 1. The vertices of these triangles are the midpoints of a, b and c, the centroids of surfaces *p−a−b*, *p−b−c* and *p−c−a*, and the centroid of the tetrahedron at 1/4(a + b + c).

Consider the pair of triangles, one joining the midpoint of a with the centroids of the adjacent sides, and the second connecting these two centroids to the centroid of the tetrahedron (shown in blue on Figure 1). Such a pair of triangles will be in the same plane.

The cross product, x, of the vectors along two sides of the triangle gives a vector normal to the surface with magnitude twice the area of the triangle. For the first triangle:

(12) $${\underline{x}}_{1}=\left(\frac{1}{3}(\underline{b}+\underline{a})-\frac{1}{2}\underline{a}\right)\times \left(\frac{1}{3}(\underline{c}+\underline{a})-\frac{1}{2}\underline{a}\right),$$

and for the second,

(13) $${\underline{x}}_{2}=\left(\text{​}\frac{1}{3}(\underline{c}+\underline{a})-\frac{1}{4}(\underline{a}+\underline{b}+\underline{c})\text{​​}\right)\times \left(\text{​}\frac{1}{3}(\underline{b}+\underline{a})-\frac{1}{4}(\underline{a}+\underline{b}+\underline{c})\text{​​}\right)\text{​}.$$

Expanding these out and collecting terms yields

(14) $${\underline{x}}_{1}+{\underline{x}}_{2}=\frac{1}{12}\underline{a}\times \underline{b}+\frac{2}{12}\underline{b}\times \underline{c}+\frac{1}{12}\underline{c}\times \underline{a}.$$

By symmetry, the other two pairs of triangles give similar expressions in which the roles of *A*, *b*, and *c* are rotated, so the sum of cross products for all six triangles,

(15) $$\underline{x}=\frac{1}{3}\underline{a}\times \underline{b}+\frac{1}{3}\underline{b}\times \underline{c}+\frac{1}{3}\underline{c}\times \underline{a}.$$

The contribution of this element, ρ_*tet*_, to the surface integral in Equation (8) is $\frac{1}{2}$ *E* · x so the total contribution from this tetrahedron is

(16) $$\rho_{tet}=\frac{1}{6}\left(E\cdot (\underline{a}\times \underline{b}+\underline{b}\times \underline{c}+\underline{c}\times \underline{a})\right).$$

Substituting *E* = ∇ Φ allows *ρ* to be calculated in terms of the potentials at the vertices. For surface elements that have a capacitative boundary, th capacitance *C*_*p*_ is just the sum of contributions from surface triangles with a vertex at *p*. The rate of change of the potential satisfies

(17) $$\begin{array}{l} C_{p}\frac{\partial \Phi_{p}}{\partial t}=\int_{surface}\sigma E\cdot \underline{n}\\ \;=\sum_{triangles}\sigma E\cdot {\underline{n}}_{tr} \end{array}$$

where the sum is taken over the triangles forming the surface to element *p* as above.

For each vertex this yields an equation of the form

(18) $$C_{p}\frac{\partial \Phi_{p}}{\partial t}=\sum_{q}G_{p,q}(\Phi_{q}-\Phi_{p})$$

where the sum is over the neighbors, *q*, of *p* and the quantities *G*_*i, j*_ are constants representing the geometry of the mesh. They can be computed programatically by gathering together the terms involving each neighboring vertex in Equation (17).

#### 2.1.3. Solution method

The unknowns are the potentials at vertices of the grid mesh and the equations are of the form:

(19) $$C\frac{dV}{dt}=\sum_{i}G_{i}(Vi-V)+I$$

where *I* is the imposed current and *C* is zero for internal elements.

Over a time interval δ*t* during which *dV/dt* can be treated as constant, this gives algebraic equations connecting the potential at time *t*+1 with the potentials at time *t*:

(20) $$C(V_{t+1}-V_{t})=\sum_{i}G_{i}(V_{i,t+1}-V_{t+1})+I\delta t.$$

Collecting the time terms together:

(21) $$\left(C+\delta t\sum G_{i}\right)V_{t+1}-\delta t\sum_{i}V_{i,t+1}=CV_{t}+I\delta t.$$

Equation (21) is of the form

$$\sum_{j}M_{i,j}V_{j,t+1}=R_{i}$$

in which the matrix M is constant over a timestep and the right hand side contains terms from any applied currents.

At each step, the solution process involves iterating over the mesh to populate *M* and *R*, and then solving the matrix equation to compute the potentials. The matrix element *m*_*i, j*_ of *M* is only non-zero if the vertices *i* and *j* are neighbors. However, although the matrix is initially sparse, direct solution methods such as Gauss–Jordan elimination involve populating more elements that were originally zero. The extent to which this occurs depends how the elements are ordered. For a computationally efficient solution it is therefore important to find an ordering of the mesh points that minimizes infill during the matrix solution. In the present study two approaches have been explored. In the first, the mesh points are ordered according to their position along the principal axis of the structure being modeled. As long as the structure has one axis which is significantly longer than the other, this keeps the non-zero elements of *M* close to the diagonal (see Results). An upper bound can be placed on the number of elements each side of the diagonal that must be stored thereby reducing total memory requirements compared to a full matrix solution. For more complex shapes a breadth-first tree iteration over the mesh visiting each point only once, with a search for the best starting point included, gives better results in terms of memory bounds and total operation count (see Results).

### 2.2. Integration with stochastic reaction-diffusion simulation in steps

An E-Field implementation is included in STEPS 2 and integrated with spatial reaction-diffusion simulations on unstructured tetrahedral meshes that accurately represent cellular morphology. A model that is built in STEPS, in which tetrahedral subvolumes are linked with diffusive molecular flux, may be simulated stochastically, or converted to a set of ordinary differential equations that are then solved deterministically in CVODE (Cohen and Hindmarsh, 1996). The addition of the E-Field object brings with it several new components to STEPS models. These objects allow simulation of effects that occur in excitable membranes, such as voltage-dependent channel transitions and ligand binding, along with channel currents. The objects that have been added in version 2 of STEPS since version 1.3 (Hepburn et al., 2012) are:

- Membrane (class steps.geom.Memb): Represents the membrane across which the electrical potential will be solved. It is comprised of a collection of triangles specified by one or more patches (class steps.geom.TmPatch), which must form a single surface that can, however, be open (i.e. it may contain holes) or closed. The support of closed loops and therefore torus topologies is relevant for many cell biological cases. The Membrane can be on the surface of the tetrahedral mesh, or may be an internal surface so as to allow outer compartments. Currently only one Membrane may exist in a STEPS simulation (though see Future improvements to simulation realism).
- Channel (class steps.model.Chan): Used to represent a specific type of chemical species: one that can undergo conformational changes, which may be voltage-dependent. In practice, in STEPS models a Channel object exists only to group a set of Channel States.
- Channel State (class steps.model.ChanState): Used to model one specific configuration of a Channel. Channel States are similar to Species objects in STEPS in that they may diffuse in volumes, or be bound to surfaces, but with the important difference that a Channel State may be defined as a conducting state with the mapping of currents (presently of class steps.model.GHKcurr or steps.model.OhmicCurr) to that state. A Channel State may only undergo voltage-dependent transitions and conduct currents when embedded in a Membrane and not when diffusing in a volume (though they may undergo ordinary reactions when in any location). Channel States are used as the basis of Markov-schemes in STEPS, and may interact with surrounding intra- and extra-cellular compartments, allowing for features such as phosphorylation-dependent modulation of channel conductance.
- GHK Current (class steps.model.GHKcurr): Describes a current passing through a given Channel State which is approximated by the Goldman-Hodgkin-Katz flux solution to electrodiffusion (Goldman, 1943; Hodgkin and Katz, 1949). The GHK equation has been shown to be an accurate approximation to full electrodiffusion under a wide range of physiological conditions, only breaking down when channel pore occupation saturates or competition occurs between different ionic species [see Hille, 2001a for further detail on the simplifying assumptions and discussion of the accuracy of the GHK equation]. Fluctuations in ionic concentrations both around and within a channel pore can result in significant noise in single-channel current (Mak and Webb, 1997), indicating the need for stochastic currents and sampling of local concentrations around each individual channel. The ionic flux in STEPS is calculated stochastically and discretely within the reaction-diffusion computations, with a rate derived from local concentrations around each channel, and this flux (optionally) results in transport of ions between compartments (see GHK Current calculation). This is particularly useful when computing the flux of important signaling ions, such as calcium, to a good degree of accuracy.
- Ohmic Current (class steps.model.OhmicCurr): Represent a channel current as an Ohmic current, and are therefore defined by a single-channel conductance and reversal potential. This current does not relate to a transport of ions between compartments in a STEPS simulation. Ohmic Current objects are included due to their prevalence in many models (for example in Hodgkin–Huxley models) and for considerations of efficiency, but for many channels the GHK Current should give a more accurate representation of the true biophysical current (see GHK flux as a more accurate representation of single-channel currents compared to the Ohmic approximation).
- Voltage-Dependent Surface Reaction (class steps.model.VDepSreac): Used to model processes that take place on a Membrane where the reaction propensity depends on the local potential across that surface. Such processes are used for modeling channels that undergo voltage-dependent conformational changes [e.g., sodium and potassium channels in Hodgkin-Huxley models, calcium-activated potassium channels (Anwar et al., 2013)] and/or voltage-dependent ligand binding [e.g., models of NMDA receptor voltage-dependent channel block by magnesium (Vargas-Caballero and Robinson, 2004)]. A channel may of course undergo both voltage-dependent interactions as well as non-voltage-dependent ones such as in Vargas-Caballero and Robinson, 2004 and the mslo/BK-type channel in Anwar et al., 2013, which undergoes voltage-dependent conformational changes and non-voltage-dependent calcium-activation.

### 2.3. STEPS simulation algorithm

**Figure 2:**
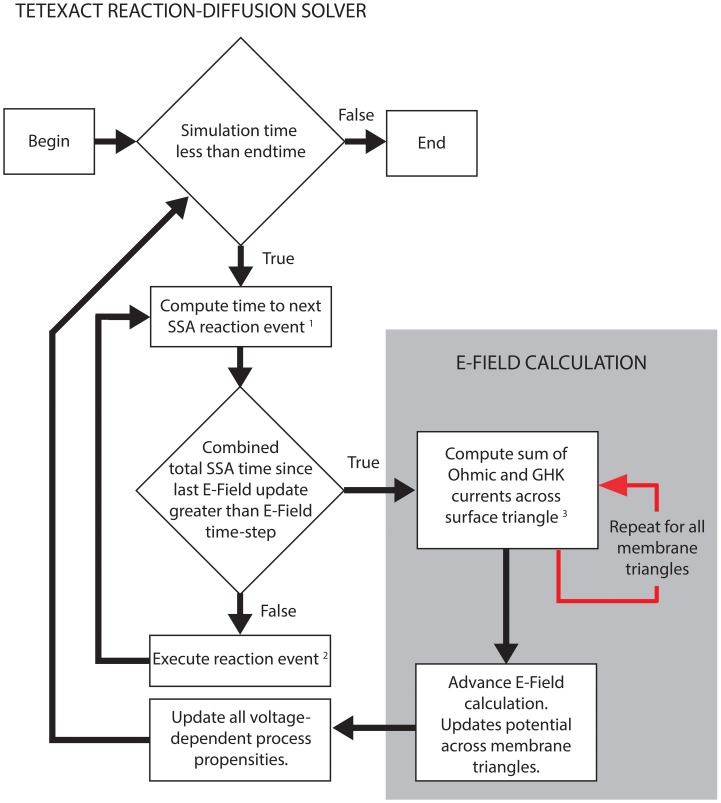
**STEPS simplified algorithm of spatial stochastic simulation including an excitable membrane**. The reaction-diffusion calculations in Tetexact and the E-Field membrane potential updates are performed separately, but are integrated frequently (typically every 0.01 ms of simulation time). This time-step for the integration allows several SSA steps to occur between communications, and is adaptive to align exactly with the SSA clock. The communication consists of membrane currents stored by Tetexact being input to the E-Field calculation, which computes the new mesh potentials that are input to Tetexact to update voltage-dependent processes. (1) SSA queue includes voltage-dependent surface reactions and GHK current single-ion events. (2) If the event is a GHK current single-ion event, the charge of the ion that passes through the channel is added to the total charge of all ions for that membrane triangle since the last E-Field update. Every membrane triangle stores a total charge. (3) The total charge from GHK single-ion events is divided by the E-Field timestep and combined with Ohmic currents to calculate total current for each membrane triangle per timestep.

Figure 2 shows the simplified algorithm for the E-Field implementation in STEPS. The reaction-diffusion calculation and the E-Field calculation run in series, but communicate often by a user-defined time-step. The SSA simulates the exact time of each reaction, updating its internal clock every time a reaction is executed, and so time-steps are not regular. The communication time is adapted to occur exactly in line with the SSA time-clock, so the E-Field calculation will usually be performed at a slightly lower time-step than was specified, and never longer. This approach leaves the SSA clock independent and so does not affect the accuracy of the reaction-diffusion computation. The intention is that the model will provide an SSA mean time-step that is much smaller than the E-Field time-step to give many SSA updates between each communication, but the implementation will still work even if this is not the case.

### 2.4. GHK current calculation

The GHK Current object in STEPS 2 calculates ionic channel flux by the GHK flux equation (Hille, 2001a) in single-channel form:

(22) $$I_{s}=P_{s}z_{s}^{2}\frac{V_{m}F^{2}}{RT}\frac{{\left[S\right]}_{i}-{\left[S\right]}_{o}exp(-z_{s}V_{m}F/RT)}{1-exp(-z_{s}V_{m}F/RT)}$$

where *I*_*s*_ is the single-channel current of ion S (amps), *P*_*s*_ is the single-channel permeability of ion S (*m^3^*.*s^−1^*), *z*_*s*_ is the valence of ion S, *V*_*m*_ is the membrane voltage (volts), F is the Faraday constant, R is the gas constant, T is temperature (kelvin), [*S*]_*i*_ is the intracellular concentration of ion S (mol.*m*^−3^) and [*S*]_*o*_ is the extracellular concentration of ion S (mol.*m*^−3^).

To simulate a GHK current in STEPS, a GHK current object is created internally for every mesh triangle that represents a section of the Membrane. Each triangle calculates the single-channel current based on Equation (22) by the fixed user-supplied simulation parameters of single-channel permeability, ion valence and temperature, the variables [*S*]_*i*_ and [*S*]_*o*_, which are sampled from the inner and outer tetrahedrons to that triangle, respectively, and the local membrane potential. Since the value of these variables can be different for every membrane triangle each triangle can store a unique local value for this flux. The calculated current is then divided by the amount of elementary charge for one ion (e.g., 2e for Ca^2+^) to give the flux as a number of molecules to pass through the channel per second. GHK currents are mapped to a Channel State and so the transport rate is multiplied by the population of the Channel State to give a total rate for this “reaction.” This is then treated as an ordinary “reaction rate” in STEPS and is solved within the SSA for one reaction-diffusion time-step (Figure 2). When this “reaction” executes it results in transport of ions between compartments (e.g., from an extracellular tetrahedron to an intracellular tetrahedron or vice-versa) and the rate is re-calculated due to the changes in concentration around the local membrane surface. A simplified GHK current object is also available, which is a pure current that does not model ion transport. Per reaction-diffusion time-step STEPS stores, triangle by triangle, the total charge that crossed the membrane triangle. At the end of a time-step these numbers are converted to currents, added to any other GHK currents and Ohmic currents, and passed to the E-Field object to update potential. Single-channel currents are then recalculated with the new V, transported charges reset to zero and the process begins again.

## 3. Results

### 3.1. Validations

To demonstrate the accuracy of the E-Field method, we performed the Rallpack tests (Bhalla et al., 1992) using tetrahedral meshes to represent the cable compartments. The Rallpack tests are a set of benchmarks that were designed to evaluate the accuracy of neuronal simulators. Although the Rallpacks are based on somewhat larger compartments than those to which the E-Field method was intended to be applied, these compartments can be supported in tetrahedral meshes and are useful for accuracy studies.

#### 3.1.1. Rallpack1: uniform unbranched passive cable

A mesh of 220,615 tetrahedrons was generated to represent a cylinder of length 1 mm and diameter 1 μm. The mesh was controlled to give an almost perfect match in volume to a true cylinder, but contained an unavoidable error of 1.0% in surface area. Membrane resistance and capacitance were controlled to match the values for cylindrical geometry perfectly.

The current of 0.1nA was injected over the nodes at one end of the cylinder, and potential recorded at both ends (0 and 1000 μm). The E-Field calculation was performed every 0.01 ms up to total simulated time of 0.25 s.

**Figure 3:**
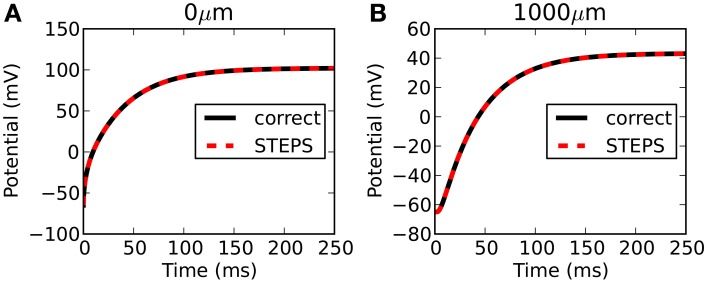
**Rallpack 1 results**. Output from the E-Field method implementation in STEPS on the mesh of 220,615 tetrahedrons is compared to the correct solution at both ends of the cylinder (**A**: 0 μm and **B**: 1000 μm).

STEPS output is highly accurate with respect to the correct solution. Figure 3 compares the output from STEPS to the correct solution at 0 μm (Figure 3A) and 1000 μm (Figure 3B). The RMS difference was 0.0102 mV at 0 μm and 0.0095 mV at 1000 μm, the small error likely to originate from the fact that the curved cylindrical surface cannot be represented perfectly with a tetrahedral mesh or from discrepancies between the 1D approximation and the 3D solution.

**Figure 4:**
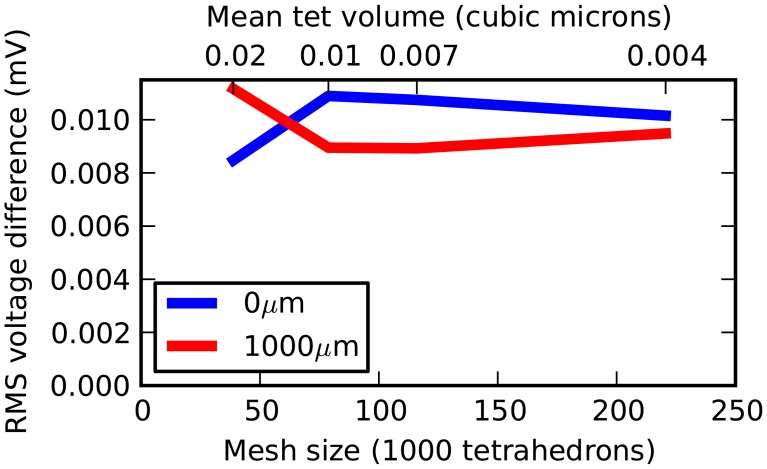
**Rallpack 1 simulation accuracy as a function of mesh size**. The RMS voltage difference between output from the E-Field method implementation in STEPS and the correct solution at both ends of the cylinder (0 and 1000 μm) at 4 different mesh sizes of 38,819, 78,778, 115,979, and 220,615 tetrahedrons.

Three further meshes were tested representing the same geometry but with 38,819, 78,778, and 115,979 tetrahedrons. All meshes were generated with a near-perfect match in volume to a true cylinder, with unavoidable errors of 3.6%, 2.6% and 2.0%, respectively, in surface area. Figure 4 shows a plot of mesh size (number of tetrahedrons) vs. RMS accuracy. Despite decreasing accuracy of true cylindrical geometry representation with fewer tetrahedrons, all meshes produced highly accurate results with respect to the correct solution. This demonstrates that, in this case, the coarser meshes can reliably be used for simulations, an important consideration for runtime (see Efficiency).

#### 3.1.2. Rallpack2: branched passive cable

The full geometry description for Rallpack2 is 10 levels of symmetric branching following the 3/2 power law (Rall, 1959) to test the ability of simulator to handle branching. While this geometry is easy to represent mathematically, it was not possible to generate a suitable tetrahedral mesh. This is due to the very large difference in length and diameter for the first level (1 compartment of length 32 μm diameter 16 μm) compared to the tenth level (512 compartments of length 4 μm and diameter 0.25 μm). The 3/2 power law, which means the two daughter branches have a total diameter approximately 26% larger than the parent branch, also introduces complications. However, it was possible to generate a mesh of 8 levels (drawing shown in Figure 8), with daughter branch faces attached to as large an area of the parent branch faces as possible. This mesh contained 60,066 tetrahedrons and was controlled to give a perfect match in volume to the ideal geometry, with a 4% error in surface area.

The current of 0.1 nA was injected over the nodes at one end of the structure, and potential recorded at both ends (0 μm and approximately 130 μm). The E-Field calculation was performed every 0.01 ms up to total simulated time of 0.25 s.

**Figure 5:**
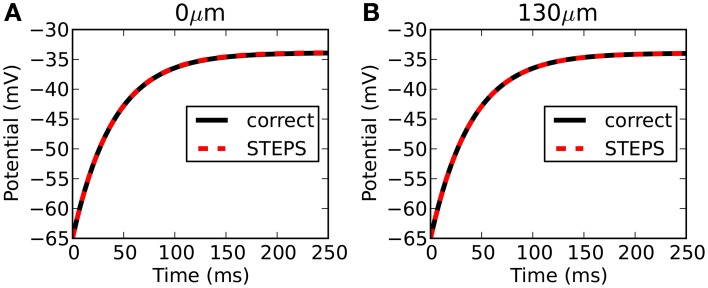
**Rallpack 2 results**. Output from the E-Field method implementation in STEPS on the mesh of a branched dendritic structure of 8 levels is compared to the correct solution at both ends of the cable (**A**: level1, 0 μm and **B**: level8, 130 μm).

Figure 5 compares the output from STEPS to the correct solution at 0 μm (Figure 5A) and 130 μm (Figure 5B). The RMS difference is 0.044 mV at 0 μm and 0.002 mV at 130 μm. Due to mesh-generation constraints it was not possible to test other meshes with different numbers of tetrahedrons.

#### 3.1.3. Rallpack3: hodgkin-huxley model

Since there is no analytical solution for Rallpack3, tests involved comparison to benchmark simulations in the deterministic cable-equation solver NEURON (Hines and Carnevale, 2001), version 7.3. All simulations in both STEPS and NEURON were run on a Mac Pro with 2.66 GHz Intel Xeon processor and 16 GB 1066 MHz memory under similar conditions.

**Figure 6:**
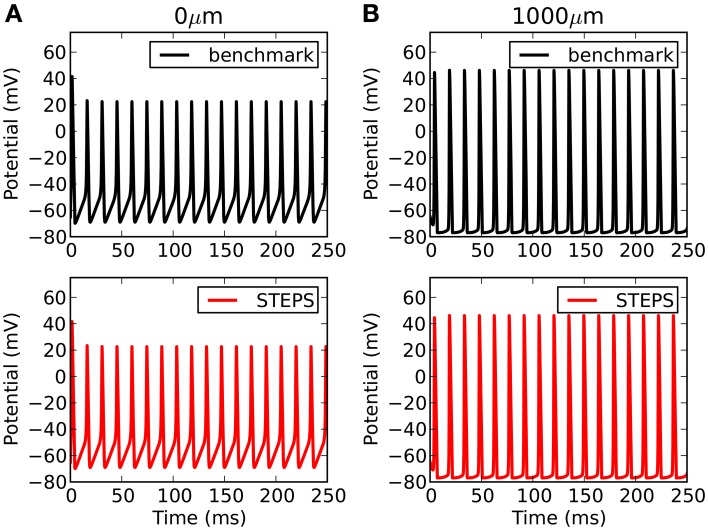
**Rallpack 3 results**. The benchmark in STEPS described in the text is compared to the benchmark solution from NEURON at both ends of the cable (**A**: 0 μm and **B**: 1000 μm).

Comparison to another simulator raises the opportunity not only to benchmark accuracy, but also performance. Such comparisons are complicated by the fact that there are many factors affecting performance over orders of magnitude in both simulators. For NEURON this includes the number of segments, the solution method (backward Euler or CVODE), time-step (backward Euler solution) or tolerance (CVODE solution). For STEPS in solver TetODE this includes the mesh size, CVODE tolerance and integration time step. Furthermore, the Rallpack3 test must be run in deterministic solver TetODE, yet STEPS is intended to be mostly applied to stochastic simulations which have different runtime considerations (Hepburn et al., 2012). However, in an attempt to benchmark performance in deterministic mode, through rigorous tests we chose as a benchmark in each simulator the approximate point at which both simulators reach very high accuracy with close agreement between the two simulators, and less than a 1 mV RMS difference with any order of magnitude increase in factors that improve accuracy (smaller time step, more segments, more detailed meshes etc). For NEURON these conditions were with a backwards-Euler time-step of 1 μs and 1000 segments. For STEPS the conditions were an equivalent cubic mesh of 1135 tetrahedrons, CVODE tolerances of 10^−6^ and integration time-step of 5 μs. The runtimes under these highly-accurate conditions were 703 s in NEURON and 847 s in STEPS. It is possible to improve performance in both simulators compared to the benchmark conditions, although this will come at some cost to accuracy. Figure 6 compares the benchmark simulations in STEPS and NEURON. Accuracy was measured by comparing RMS voltage over the entire time range of 250 ms and the mean time difference between the spike peaks, at both ends of the cable (0 and 1000 μm). The RMS differences were 0.17 and 0.90 mV and spike timing differences were 2.5 μs and 23 μs, at the 0 and 1000 μm ends, respectively. To put this accuracy into context the differences between this benchmark with a 1 μs time-step and a 5 μs time-step using the NEURON simulator are 3.0 mV, 5.4 mV, and 100 μs, 100 μs at the 0 μm and 1000 μm ends, respectively.

**Figure 7:**
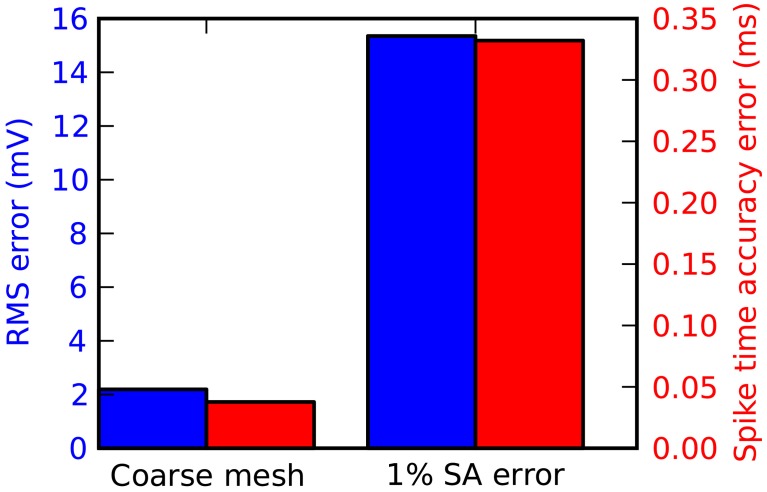
**Tetrahedral mesh effects on accuracy of the Rallpack3 test**. Measurements taken relative to the benchmark at the 1000 μm end of the cable. Reduction in accuracy relative to the benchmark measured by RMS voltage difference (blue) and mean spike-time resolution (red) for a coarse mesh of 600 tetrahedrons (left bar) and error of 1% in surface area (right bar).

For most applications in STEPS, particularly stochastic models, mesh generation is the most important consideration for accuracy. Accuracy can suffer if tetrahedrons are too large, and in addition mesh quality affects accuracy particularly when there are discrepancies compared to ideal surface area and volume. Figure 7 demonstrates when there is a loss of accuracy relative to the Rallpack3 benchmark due to these effects. Reducing the number of tetrahedrons to 600 from 1135 (i.e., increasing mean volume from 0.7 μm^3^ to 1.3 μm^3^) results in small loss of accuracy (Figure 7: left bar). All meshes with more than 1135 tetrahedrons were accurate. In comparison to the error from large tetrahedrons, an introduced increase in surface area of just 1% gives a much larger loss of accuracy (Figure 7: right bar) demonstrating that it is essential to compensate for errors in membrane surface area in generated meshes.

In general for applications, it is important that the many factors affecting accuracy are taken into account in both stochastic or deterministic simulations, and that an acceptable accuracy is chosen relative to runtime considerations. In addition a few percent error in surface area compared to perfect cylinders, often unavoidable in tetrahedral meshes, can result in a significant loss of accuracy, and so it is desirable to account for this error by applying corrections to membrane properties.

### 3.2. Efficiency

**Figure 8:**
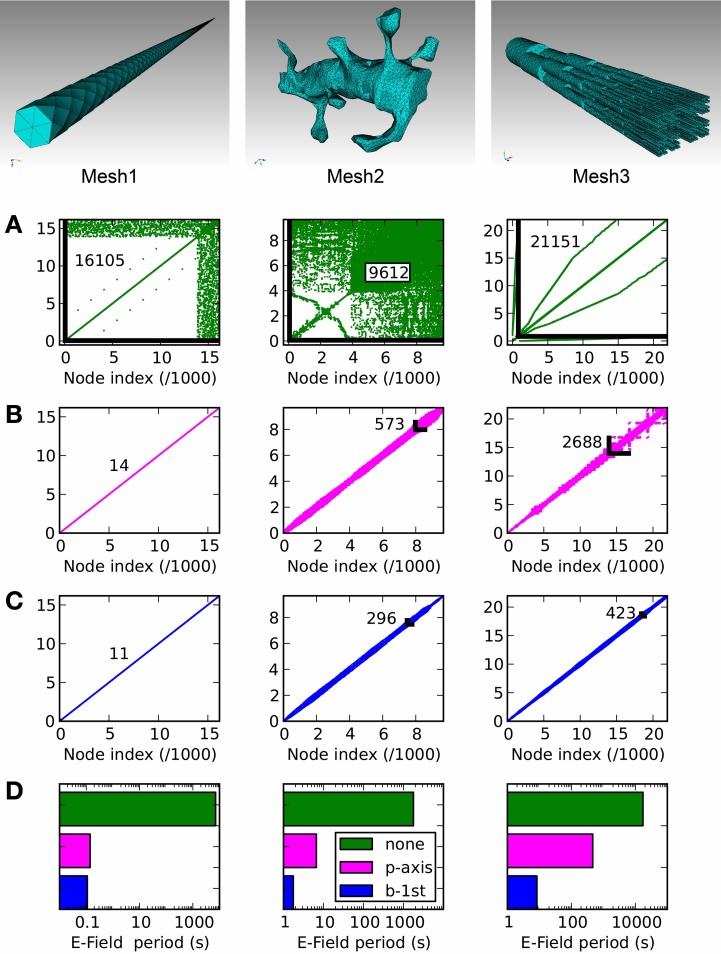
**Node connectivity and its effect on E-Field calculation period**. Connectivity matrices are displayed for three tetrahedral meshes of different geometries, with **(A)**: no ordering (mesh generator output), **(B)**: principal axis ordering, **(C)**: breadth-first search ordering. The maximum node separation is displayed for each connectivity matrix as a number and, where visible, a black line showing the maximally separated node connection. **(D)**: The E-Field calculation performance shows a strong dependency on the ordering method, with the application of one of the two ordering methods always producing a strong reduction in calculation period compared to the unordered case (green). The benefit of the breadth-first search (blue) compared to principal axis ordering (magenta) depends on the mesh geometry, with a significant improvement for Mesh 2 and a stronger benefit for Mesh 3.

**Figure 9:**
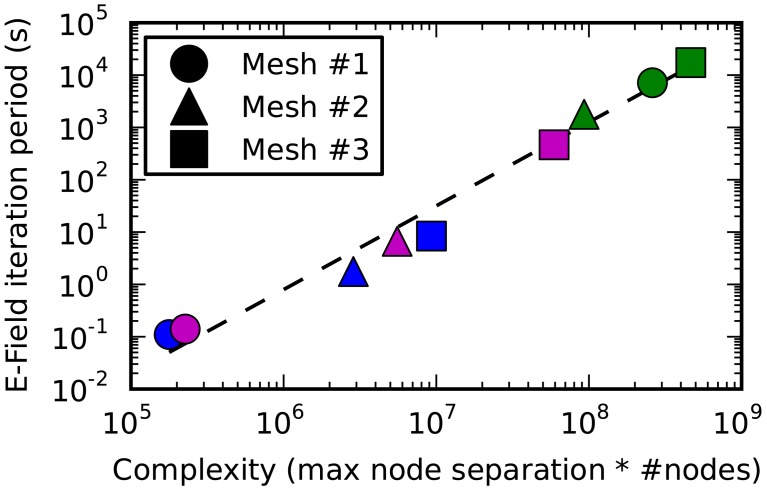
**The E-Field iteration period vs. calculation complexity**. The E-Field iteration period results for the 3 meshes shown in this figure are plotted as a function of the calculation complexity, which is estimated as the maximum node separation multiplied by the total number of vertices. This relationship appears to approximately follow a power law, and the function y = 2^*^10^-10^.*x*^1.6^ is shown by a dashed line. The calculation period for breadth-first ordering (blue) gives a small maximum node separation that gives sub 10 s performance for all the tested meshes, and the principal axis ordering (magenta) performs comparably for Mesh 1 and 2, but gives a large maximum node separation for Mesh 3 resulting in a large iteration period (magenta square). Raw ordering (green) always gives large node separations that result in unworkably long E-Field iteration periods.

In Figure 8 we present three example tetrahedral meshes, and discuss how these meshes can be optimized for computations. As mentioned in E-Field: the tetrahedral mesh potential calculation in STEPS, re-ordering of mesh nodes (vertices) is essential to achieve an efficient solution. This amounts to minimizing the largest index difference between connected nodes, and because meshes in raw form are usually not sorted in this way (Figure 8A) it is essential that a sorting method is applied. We tested two sorting methods, which are available in STEPS 2: principal axis ordering (Figure 8B) and a breadth-first search (Figure 8C). The important number to measure in order to determine the performance of the solution for any applied sorting method is the resulting maximum difference between node indices of all connected nodes in the mesh, because a larger number gives a slower computation (Figure 9). Mesh 1, a long cylindrical structure, shows the most startling benefit to performance by application of a sorting method, resulting in a decrease in node index separation from 16,105 in the unsorted case (which is close to the total number of nodes: 16,177) to 14 with principal axis ordering and 11 with breadth-first search, a 3-order of magnitude decrease, which reduces the period for E-Field iteration from several thousands seconds to 0.14 and 0.11 s, respectively (Figure 8D). This also demonstrates that the principal axis ordering method is suitable for structures with one long axis with only a small difference between that and the breadth-first search for this mesh. Mesh 2 contains more complex realistic geometry: a reconstruction of a spiny dendrite from CA1 stratum radiatum (http://synapses.clm.utexas.edu/anatomy/ca1pyrmd/radiatum/k24/k24.stm). For this mesh the ordering methods produce a 2-order of magnitude maximum node separation reduction, though there is a significant benefit in choosing the breadth-first search, which produces an E-Field period of 1.76 s compared to 6.65 s for principal axis ordering. Mesh 3, which represents the branched dendrite used for Rallpack2, shows the greatest benefit for the breadth-first search over principal axis ordering. With an unordered maximum node index separation of 21,151, principal axis ordering only reduces this to 2688 compared to 423 for the breadth-first search, resulting in an unacceptably high E-Field period of 465 s for principal axis ordering, but a manageable 8 s for the breadth-first search. The breadth-first search is a slower search compared to principal axis ordering, so for morphologies where the result is similar it is favorable to apply principal axis ordering. For example the 100 % breadth-first search (testing all starting nodes) took 146 s for Mesh 2 and 736 s for Mesh 3 whereas principal axis ordering typically only takes a few seconds. However, for these meshes, the gains to performance with a breadth-first search outweigh the cost of the initial search, and in addition vertex ordering may be saved to file in STEPS so that the sorting method need only be applied once. Practically, version 2.1 of STEPS allows fewer than 100% of starting points to be visited benefitting search time with only a small cost to performance, and includes the Cuthill-McKee improvement to the standard breadth-first search (Cuthill and McKee, 1969). All simulations were run on a Mac Pro with 2.66 GHz Intel Xeon processor and 16 GB 1066 MHz memory.

Figure 9 shows how the performance of the E-Field calculation depends on the calculation complexity, which is represented by the product of the maximum node index separation with the total number of nodes since this defines the size of the band diagonal matrix at the core of the E-Field calculation (see Solution method). The relationship appears to approximately follow a power law: *Period ∝ Complexity^1.6^*. This power law can be used to estimate the E-Field period when running a STEPS simulation, and demonstrates the need to find the smallest maximum node separation.

## 4. Discussion

The E-Field method implemented in STEPS 2, while validated against 1D cable-like solutions and benchmarks, automatically allows membrane potential to be solved in any geometry that can be represented by a tetrahedral mesh, which allows very close 3D morphological precision (Hepburn et al., 2012). This can be very important when considering the complex neuronal morphology in which key signaling systems occur where a 3D solution is required. Although 1D cable-like solutions give reasonable accuracy in whole-cell simulators, for the detailed level of cellular geometry surrounding typical reaction-diffusion systems the errors introduced by simplifying complex morphology into a 1D cable-like solution can be non-negligible (Lindsay et al., 2004). Closely tied to this consideration is also the need for close coupling between local membrane potential and local excitable membrane effects such as voltage-dependent gating and stochastic channel currents (Mak and Webb, 1997), all of which are supported by our approach but are difficult to achieve by mapping separate solvers together.

Our method, while offering significant gains in simulation accuracy of the reaction-diffusion systems in and around excitable membranes, is itself an approximation to full electrodiffusion. The obvious benefits of using the E-Field method compared to full electrodiffusion are of efficiency, where full electrodiffusion would be unworkably slow. Inclusion of the GHK equation incorporates the most significant electrodiffusion effects, although our approach ignores the Lorentz force acting on charged ions in the presence of an electric field, as well as the component of the axial current that is carried by simulated ions. However, these effects are expected to be small because under realistic physiological conditions thermal velocities are orders of magnitude larger than drift velocities in an electric field (Hille, 2001b), and therefore this simplification will not appreciably affect simulation results (De Schutter, 2010).

### 4.1. Accuracy

The accuracy of the E-Field method has been demonstrated with established cable-equation models, importantly showing some dependence of accuracy on tetrahedron size. Tetrahedron size can be an even more important consideration in realistic reaction-diffusion applications. As well as falling within a certain size window for sufficient accuracy (Hepburn et al., 2012), there may be other important model-dependent considerations, such as localization in microdomains that exist in a narrow concentration range that will be influenced in simulations by tetrahedron size (Anwar et al., 2013). Therefore, accurate mesh generation is an important consideration for complex models. Accuracy can usually be ensured with a careful, methodological approach, which may involve benchmarking deterministic tests in a range of meshes that are close to acceptable resolution against another solver (Anwar et al., 2013). In addition, the method currently doesn't allow extracellular fields (Buzsáki et al., 2012), and so is suitable for systems where the earthed-bath assumption is a good approximation, losing some accuracy when this is not the case. It would be possible to allow for explicit solution of the extracellular space by the same method with appropriate boundary conditions, and this is a possible future addition.

### 4.2. Efficiency

Application of an ordering method is essential to achieve efficient calculations. We conclude that principal axis ordering, which has the benefit of being a quick search, is sufficient for simple shapes where one axis is much longer than the others. For complex shapes the breadth-first search should be applied. Although this is a slower search, for every mesh this search only needs to be applied once because, in STEPS, optimal indexing may be saved to a file which can be used to load the optimal indexing from for all future simulations using that mesh.

### 4.3. GHK flux as a more accurate representation of single-channel currents compared to the ohmic approximation

Ohmic currents are commonly used in computation models of neural systems, yet Ohmic currents are only a good approximation when the ionic concentrations in the two compartments separated by a membrane are quite similar, and only then is the single-channel conductance approximately constant over the relevant voltage range. When there is a significant concentration gradient this effectively means that the single-channel conductance has a voltage-dependence that is usually better described by the GHK flux equation (Hille, 2001a; Clay, 2009). There are occasions when an Ohmic current is a good approximation even when a significant concentration gradient exists, which is caused by some external effect, such as the squid giant axon sodium current which is linearized in the physiological range by partial block of the current by calcium and magnesium ions (Vandenberg and Bezanilla, 1991). However, the GHK equation is usually the more accurate representation, and this accuracy is particularly important when calculating the flux of ions that then undergo important intracellular processes, such as calcium (De Schutter, 2010).

### 4.4. Future integration with whole-cell solvers

The E-Field implementation in STEPS was developed with the intention of being applied to the complex morphologies surrounding micrometer cellular signaling regions. Although it is capable of solving membrane potential in anything that can be represented by a tetrahedral mesh and therefore could, in theory, be used in whole-cell models, often such a level of detail is not necessary and would introduce runtime concerns at such scales, and the whole-cell calculations would be much better solved by cable-theory based simulators such as PSICS (http://www.psics.org), NEURON (http://www.neuron.yale.edu) or GENESIS (http://www.genesis-sim.org). Therefore, important future work will involve integrating STEPS with one or more whole-cell and network simulators, where STEPS simulates a section of the morphology completely and the rest of the cell is solved by a whole-cell simulator, with coupling consisting ideally of a single axial current. This could be achieved in a number of ways. For example, the tools are already in place to form a connection through Python of STEPS to other simulators with a Python interface, which are many. However, this would most likely be the least efficient approach. More efficient coupling could be achieved through software designed for this goal, such as MUSIC (Djurfeldt et al., 2010), or through a more direct approach. With successful integration between simulators achieved many more applications will open up in which STEPS could potentially form a component in full multi-scale models.

### 4.5. Future improvements to simulation realism

While the introduction of accurate calculation of the time-varying electrical potential in complex 3D geometries is an important advance in multiscale simulation by making it possible to tightly integrate local membrane potential and currents with reaction-diffusion calculations, there is the possibility for further additions to this in the future. Small deviations from the GHK flux are possible in some biological channel currents; for example competition may exist in the pore which results in some weak voltage and/or concentration dependence to permeability (Jatzke et al., 2002). Improvements to calculation accuracy for such channel currents could be possible by simulating the current through pores as particles “hopping over” energy barriers (Hille, 2001a), though this could come at a significant cost to simulation runtime. In addition, the present implementation is restricted to simulating the potential across only one cellular membrane for which the outer potential is assumed fixed, yet cells can contain internal membranes (such as the endoplasmic reticulum) that are capable of charge separation and therefore contain a potential across them (Shemer et al., 2008), and can themselves contain channels and pumps that transport ions from intracellular stores (Szewczyk, 1998; Hille, 2001c) by processes that can also be voltage-dependent (Sepehri et al., 2007). Therefore, there could be many useful applications of an extension that allows simulation of the potential across such internal membranes, which will involve a modification to the present implementation to allow a varying outer compartment potential.

## Availability

STEPS is available at: http://steps.sourceforge.net

## Conflict of interest statement

The authors declare that the research was conducted in the absence of any commercial or financial relationships that could be construed as a potential conflict of interest.
